# Multi-robot replication of ant collective towing behaviours

**DOI:** 10.1098/rsos.180409

**Published:** 2018-10-17

**Authors:** Sean Wilson, Aurélie Buffin, Stephen C. Pratt, Spring Berman

**Affiliations:** 1 School of Electrical and Computer Engineering, Georgia Institute of Technology, Atlanta, GA 30308, USA; 2 Life Science, Mesa Community College, Mesa, AZ 85202 USA; 3 School of Life Sciences, Transport and Energy, Arizona State University, Tempe, AZ 85287, USA; 4 School for Engineering of Matter, Transport and Energy, Arizona State University, Tempe, AZ 85287, USA

**Keywords:** ants, swarm robotics, decentralized coordination, self-organization, reinforcement learning, heterogeneous teams

## Abstract

In this work, teams of small mobile robots are used to test hypotheses about cooperative transport by ants. This study attempts to explain a decrease in steady-state transport speed with increasing team size that was previously observed in the ant *Novomessor cockerelli*. Two models of one-dimensional collective towing are compared: one in which transporters with different maximum speeds pull the payload with continuous, variable forces and another in which transporters with identical speeds pull with intermittent, unsynchronized forces. A statistical analysis of ant data supports the hypothesis that ants behave according to the first model, in which the steady-state transport speed is the maximum speed of the slowest teammate. By contrast, the ant data are not consistent with the second model, which predicts constant speed regardless of team size. To verify these predictions, the ant behaviours in each model are translated into decentralized controllers and implemented on teams of two to four robots. The controller for the first model incorporates a real-time reinforcement learning algorithm that successfully reproduces the observed relationship between ant team size and transport speed. The controller for the second model yields the predicted invariance of transport speed with team size. These results show the value of robotic swarms for testing mechanistic hypotheses about biological collectives.

## 1. Introduction

Cooperative transport of large food items by ants is an impressive example of robust multi-agent coordination that is fully decentralized, scalable with the number of transporters, and effective in unknown environments with uneven terrain and obstacles. The ant behaviours that drive this phenomenon are still poorly understood [1,2]; however, coordination among transporters probably depends on indirect interactions through the food item being carried [3,4]. These interactions are an example of *stigmergy*, a mechanism by which individuals communicate through modifications to their environment (in this case, the payload). However, stigmergic behaviour is most likely not the sole factor contributing to successful transport. Coordination may also depend on the members of the transport team having some common information, such as the direction to the nest, and may rely on direct or explicit communication between transporters.

Further understanding of group food retrieval in ants can be applied toward developing control policies for cooperative transport by robotic swarms. Cooperative multi-robot manipulation of heavy payloads in unstructured environments has potential applications in disaster response and search-and-rescue operations, as well as automated construction and assembly tasks in remote environments. Previous approaches to multi-robot manipulation have relied on explicit communication between robots, leader–follower strategies, and prior information about the environment, physical properties of the payload, and configuration of robots around the payload [5–7]. In other approaches, collective transport is indirectly effected through the pushing efforts of robots that make contact with the load while responding to an external stimulus [4,8,9].

**Figure 1.**
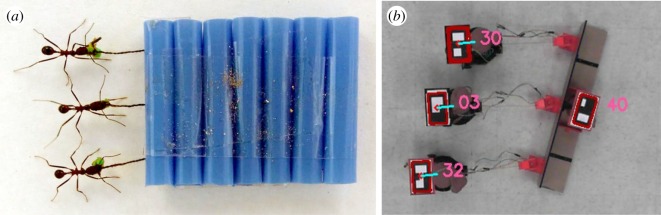
Experimental set-up for tracking collective payload towing by teams of (*a*) *Novomessor cockerelli* and (*b*) Pheeno robots.

This study considers group transport of artificial payloads by *Novomessor cockerelli*, a species of desert ant that is capable of highly coordinated, stable transport of large food items in teams [10]. An experimental study of these ants [11] found that the steady-state load transport speed decreased with increasing team size, even when the load weight per ant was held constant. The authors speculated that this effect may be caused by variation in ant orientation with respect to the payload. That is, teams usually distribute themselves along the load’s perimeter, and the force that an ant can apply may vary according to its position. For instance, a dynamical model of the ant data in [11] successfully reproduced transport dynamics by assuming that ants on the leading edge of the load pulled and lifted, while ants on the trailing edge only lifted [12]. Effects such as these might be expected to reduce speed for larger team sizes, if additional transporters are required to occupy less advantageous positions. However, this idea was not supported by a more recent experimental study in which all transporters adopted the same position [13]. This was achieved by requiring ants to move a rectangular load by pulling on strings attached to one of its sides, as shown in figure 1*a*. The results confirmed the relationship observed previously, in which load speed decreased as team size increased and *per capita* load remained constant.

Buffin *et al.* [13] also noted a large variance in the speed of single ants towing a load by themselves, suggesting that the members of transport teams vary in the maximum speeds and pulling forces they can attain. They performed simulations that assembled virtual teams from random samples of a normally distributed population of maximum individual speeds. If each team was assumed to have the speed of its slowest member, the simulations reproduced the observed decline in transport speed with team size [13]. However, that study did not examine the coordination mechanisms that would allow a team to regulate its speed in this manner.

In the present study, a multi-robot testbed is used to test hypotheses about ant group transport behaviours in order to gain insight into the observed relationship between load speed and team size [11,13]. It is assumed that all the ants in the transport team know the direction to the goal (the ants’ nest) and can navigate even while moving backwards [14], but that they have no knowledge of the load characteristics, the number of teammates or the teammates’ locations on the load relative to its centre of mass. Section 2 describes the multi-robot testbed, figure 1*b*, that was designed to mimic experiments that show a decrease in ant transport speed with increasing team size [13]. Section 3 presents two candidate models of collective towing behaviour in an effort to reproduce this observed trend. The first model assumes a team of ants with heterogeneous maximum speeds that pull on the load with continuous, variable forces, requiring the team to move at the speed of the slowest member for stable transport to occur. The second model assumes a homogeneous team of ants that pull on the load with intermittent, identical forces as they take uncoordinated steps backward toward the nest. For both models, the average steady-state transport speed is predicted and compared to the ant data. Section 4 proposes a decentralized robot controller for cooperative towing that can be implemented on a team of robots with heterogeneous maximum speeds. The controller is based on a reinforcement learning algorithm that uses only stigmergic feedback, similar to the type of information that would be available to the ants. This controller was implemented on teams of two to four Pheeno robots [15] that cooperatively towed a rectangular payload, along with a second controller that produced intermittent, uncoordinated pulling forces. Section 5 presents the steady-state transport speeds in these robot experiments and compares them to the transport speeds observed in the ant experiments. Finally, §6 discusses possible causes and advantages of the observed transport strategy in *N. cockerelli*, and §7 summarizes the results and outlines directions for future work.

## 2. Materials and methods: multi-robot experiments

**Figure 2.**
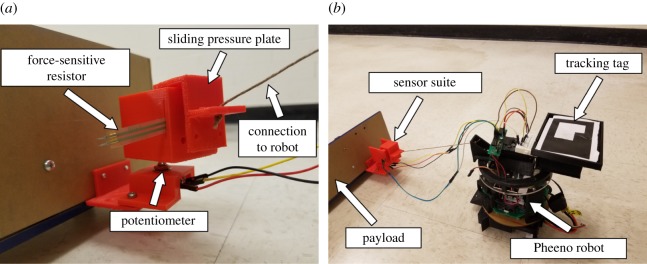
Details of the multi-robot towing experimental set-up. (*a*) A close-up, labelled view of a sensor suite attached to the payload. (*b*) A single robot connected to the payload.

Collective towing experiments were conducted with teams of two, three and four Pheeno robots. Each team pulled a load in a parallel configuration designed to mimic the arrangement of pulling ants in the experiments of Buffin *et al.* [13]. Figure 1*b* shows an overhead snapshot of the experimental set-up with three robot transporters. The payload in these experiments was a 76.2 × 10.2 × 10.2 cm L-shaped acrylic frame weighing 500 g. A three-dimensional-printed plastic basket attached to the frame allowed different masses to be added. A sensor suite was designed for each robot to measure the force vector that it applied to the load throughout the transport process. The sensor suite consisted of a three-dimensional-printed sliding pressure plate mounted on a potentiometer that allowed 270° of rotation. A circular force-sensitive resistor was used to measure the magnitude of the force that the pressure plate exerted due to the robot’s pulling force, and the potentiometer determined the direction of the force with respect to the load’s orientation. The sensor suites were affixed to the payload, and a hemp cord connected each robot to a sensor suite, as shown in figure 2. The experiments were filmed with an overhead camera (Microsoft Life Cam, resolution of 720p) at a rate of 30 frames per second. The robots and payload were marked with two-dimensional binary identification tags to enable real-time tracking of their positions and orientations by the overhead camera.

## 3. Two models of ant behaviours and the resulting payload speed

### 3.1. Ants with continuous, adaptive pulling forces

**Figure 3.**
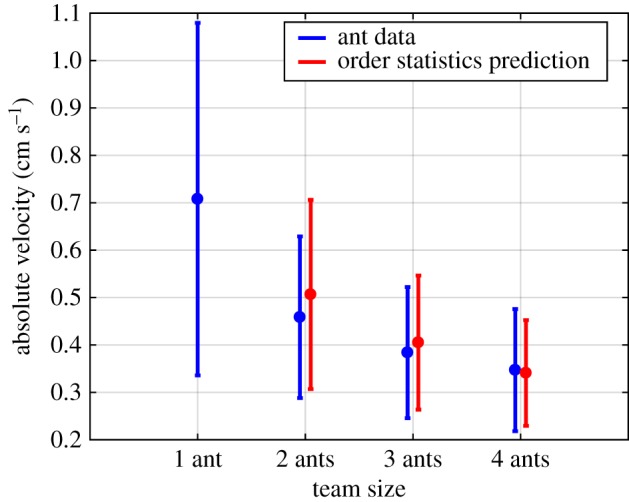
*Blue plots*: steady-state speed of an artificial load (0.3 g per ant) towed by different numbers of ants [13]. The circles with error bars represent the mean ± standard deviation across 30 experimental trials for one ant, 24 for two ants, 25 for three ants and 20 for four ants. *Red plots*: mean ± standard deviation of the first-order statistic of *n* = 2, 3, 4 samples from a normal distribution that is fit to individual ant transport speed data.

Figure 3 plots data from Buffin *et al.* [13] that show a decrease in steady-state load speed with increasing team size, even though each ant was pulling the same amount of weight. Tukey range tests showed that individual transport speed was significantly faster than transport by teams of two ants (*p*_1,2_ = 0.014), three ants (*p*_1,3_ = 1.60 × 10^−5^) or four ants (*p*_1,4_ = 6.34 × 10^−6^), but there was no significant difference among teams of different size (*p*_2,3_ = 0.70, *p*_2,4_ = 0.42 and *p*_3,4_ = 0.96). The plot also reveals a large variance in transport speed by single ants. As noted in Buffin *et al.* [13], this suggests that an ant transport team can be modelled as a heterogeneous group whose members are capable of different maximum speeds and pulling forces. Such a team would be able to cooperatively tow the load only as fast as the speed of its slowest member, requiring coordination among the teammates.

In this model, it is assumed that an ant’s maximum towing speed is distributed according to a Gaussian probability density function (pdf), *ϕ*(*v*), with corresponding cumulative density function (cdf), *Φ*(*v*) (see the electronic supplementary material). Under this assumption, a Gaussian distribution was fitted to the data in figure 3 on the steady-state towing speed of a single ant (*μ* = 0.7 cm s^−1^, *σ* = 0.36 cm s^−1^). The expected speed of the slowest member of a transport team with *N* members was then computed using order statistics [16]. The expected mean of the *r*th-order statistic from *n* samples of the distribution *ϕ*(*v*), where 0 < *r* ≤ *n*, is given by

3.1 $$E(r,n)=\frac{n!}{(r-1)!(n-1)!}\int_{-\infty }^{\infty }v{\left[1-\Phi (v)\right]}^{n-r}\Phi {\left(v\right)}^{r-1}\phi (v)\;\text{d}v.$$

The variance of the *r*th-order statistic is

3.2 $$\text{Var}(r,n)=\int_{-\infty }^{\infty }{\left(v-E(r,n)\right)}^{2}{\left[1-\Phi (v)\right]}^{n-r}\Phi {\left(v\right)}^{r-1}\phi (v)\;\text{d}v.$$

The speed of the slowest member of a team is distributed according to the first-order statistic (*r* = 1), for which equations (3.1) and (3.2) simplify to

3.3 $$E(1,n)=n\int_{-\infty }^{\infty }v{\left[1-\Phi (v)\right]}^{n-1}\phi (v)\;\text{d}v$$

and

3.4 $$\text{Var}(1,n)=\int_{-\infty }^{\infty }(v-E{\left(1,n)\right)}^{2}{\left[1-\Phi (v)\right]}^{n-1}\phi (v)\;\text{d}v.$$

To enforce realistic constraints on the range of possible ant speeds, the support of *ϕ*(*v*) was truncated to [0, *v*_max_], where *v*_max_ = 1.2 cm s^−1^ is slightly higher than the fastest towing speed measured during experiments with a single ant. The integral equations (3.3) and (3.4) were evaluated over the range of *v* ∈ [0, *v*_max_], after being renormalized to have a total probability of 1, for team sizes *n* = 2, 3, 4. The computed values of *E*(1, *n*) ± Var(1, *n*) ^1/2^ for *n* = 2, 3, 4 are plotted alongside the statistics of the ant data in figure 3. The figure shows that the mean and standard deviation of the measured towing speed for each team size are very close to those of the corresponding first-order statistics. This similarity supports the hypothesis that ant transport teams move at the speed of the slowest teammate, which implies that the ants can coordinate transport in a decentralized fashion without explicit communication, information about the payload, or knowledge of the team size and configuration.

### 3.2. Ants with intermittent, constant pulling forces

Besides the ants’ variability in speed, their unsynchronized gaits during transport could have contributed to the decrease in load speed with increasing team size. The ants in a transport team were observed to step backward while towing the load, and their out-of-phase stepping could have coincided with their application of intermittent, uncoordinated forces on the load. In this section, a dynamical model is developed to investigate the effect of this hypothetical behaviour on the steady-state transport speed.

Collective transport behaviours have previously been studied with the Kilobot robotic platform [17] and the Bristlebot, Hexapod and *μ*Tug platforms [18]. Rubenstein *et al.* [17] found that the transport speed remains the same regardless of team size when the payload mass per robot is kept constant. However, this paper did not take into account out-of-phase stepping by the robots. Christensen *et al.* [18] investigated the effect of uncoordinated stepping on effective force per robot during group towing. They found no effect for the case where the robots’ gait is non-impulsive; e.g. the case where each robot’s contact time with the ground, during which it exerts a pulling force on the load, is not extremely short compared with the stride period of its gait. Here, the dynamic model presented in [17] is combined with the gait consideration discussed in [18] to predict the effect of out-of-phase stepping on the steady-state transport speed.

During the experiments, the ants transported the load along an approximately straight path and produced very little load rotation. Hence, the load dynamics can be modelled as translation in the plane using Newton’s second law of motion:

3.5 $$\sum_{i=1}^{N}F_{i}+F_{f}=ma,$$

where $F_{i}\in R^{2}$ is the force applied by ant *i*, $F_{f}\in R^{2}$ is the kinetic frictional force on the load, *m* is the load’s mass and $a\in R^{2}$ is the load’s acceleration. Using an ideal motor assumption to relate force to velocity, the force applied by ant *i* can be modelled by the linear relation

3.6 $$F_{i}=K(v_{\max}-v_{i}\;\cdot \;\hat{x})\hat{x},$$

where *K* > 0 is a constant gain, *v*_max_ is the maximum ant speed under no load, $v_{i}\in R^{2}$ is the velocity of ant *i* and $\hat{x}\in R^{2}$ is the direction of the load transport. Under the assumption that the load is in static equilibrium in the vertical direction, the frictional force is given by

3.7 $$F_{f}=-\mu_{k}mg\hat{x},$$

where *μ*_*k*_ is the coefficient of kinetic friction of the load on the ground, and *g* is the acceleration due to gravity.

In this model, all ants move at the same speed. Since the ants pull on strings to tow the load, they are rigidly attached to the load when applying force (e.g. the ants may not move faster than the load). This model assumes that a transporting ant will not accumulate enough slack in its string such that its pulling effort does not affect the payload, as was evident in the ant transport videos. During transport, the load stops immediately when the ants stop pulling it. This allows for the use of the quasi-static motion assumption [19,20], implying that the load velocity $v_{L}\in R^{2}$ is in the same direction as the net force applied by the ants. These assumptions simplify the sum over the forces defined in equation (3.6) to,

3.8 $$\sum_{i=1}^{N}F_{i}=NK(v_{\max}-∥v_{L}∥)\hat{x}.$$

Inserting this total applied force into equation (3.5), along with the frictional force defined in equation (3.7), and solving equation (3.5) for $∥v_{L}∥$ at steady-state (**a** = **0**) yields

3.9 $$∥v_{L}{∥}_{ss}=v_{\max}-\frac{\mu_{k}mg}{NK}.$$

To include the effect of asynchronous ant gaits, the model can incorporate a probability *p*_*f*_ = *t*_*c*_/*t*_*s*_ that an ant applies a pulling force at any given time, where *t*_*c*_ is its contact time with the ground and *t*_*s*_ > *t*_*c*_ is the period of its gait, as defined in [18]. In the case where *N* ants pull in parallel with their steps beginning at independent, uniformly random starting times between 0 and *t*_*s*_, the number of transporters *n* ≤ *N* that apply force at the same time is described by the binomial distribution, *n* ∼ *B*(*N*, *p*_*f*_). Then, the average total force exerted by the ants on the load is given by

3.10 $$\sum_{i=1}^{N}F_{i}=p_{f}NK(v_{\max}-∥v_{L}∥)\hat{x}.$$

Inserting this total applied force into equation (3.5) and solving for the steady-state load velocity results in an equation similar to equation (3.9):

3.11 $$∥v_{L}{∥}_{ss}=v_{\max}-\frac{\mu_{k}mg}{p_{f}NK}.$$

What is important to note in equation (3.11) is that the term producing a slowing effect, *μ*_*k*_*mg*/*p*_*f*_*NK*, includes the load mass *m* in the numerator and the team size *N* in the denominator. Therefore, if the ratio *m*/*N* of the load mass to the team size is kept constant, as was done in the ant experiments, then the steady-state load speed should remain the same regardless of the team size, as observed in [17]. This contradicts the observed trend in load speed from the ant experiments.

## 4. Design of robot controllers for adaptive, continuous pulling

One possible method for a heterogeneous transport team to adjust to the speed of its slowest member is through learning based on stigmergic feedback; in this case, measurements of changes in the load’s rotation in response to forces applied by all the transporters. As stated in §1, it is assumed that the ants all know the direction to their nest and that they tow the load in this direction along a straight line. Under these assumptions, the ants’ configuration on the load during the experiments (figure 1*a*) would have resulted in a net zero moment on the load if all ants pulled with identical forces. Any disparities in towing force would produce differences in the ants’ maximum possible steady-state towing speeds as well as rotation of the transported load. However, significant rotation of the payload was not observed in the ant experiments after the transport reached steady state (e.g. the load oscillated slowly but did not rotate, which would have indicated that one ant was moving faster than the others). We hypothesize that the ants detect small rotations of the load or rotational forces on the load, and that they act to reduce these before they become large. We also assume that the ants do not know their location on the load with respect to its centre of mass; i.e. they have no *a priori* knowledge of how changing their speed of transport will affect the rotation of the load. Thus, they cannot be using a simple feedback mechanism to reject the load’s rotation, since they do not know before transport whether they should speed up or slow down when sensing a change in the load orientation.

**Figure 4.**
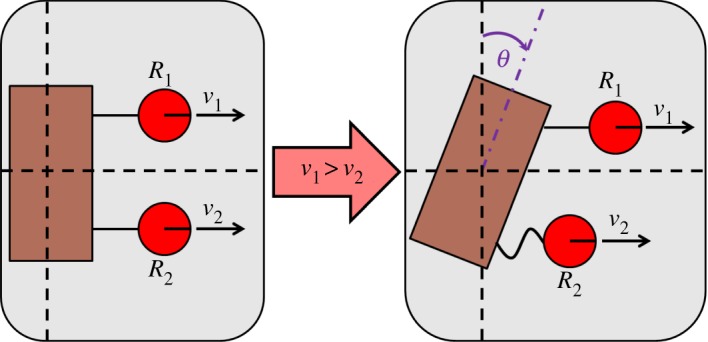
A two-robot towing scenario. Robot *R*_1_ is moving faster than robot *R*_2_, causing the load to rotate by angle θ and the towing string of robot *R*_2_ to go slack.

Ants could have implicitly communicated their differences in towing speed by measuring the collective effect of these differences on the load’s orientation and rotational dynamics during transport. To illustrate, figure 4 shows a cooperative towing scenario with two robots. Robot *R*_1_ is moving faster than robot *R*_2_, causing the load to rotate in the clockwise direction. If robot *R*_2_ were moving faster than robot *R*_1_, then the load would rotate in the counterclockwise direction. By making an association between the load’s direction of rotation and the required speed change to reject the rotation, and by measuring its own pulling force, each robot should be able to learn the conditions under which it should speed up or slow down in order to move at the same speed as the rest of the transport team. These conditions do not require the robot to know its location on the load. When there is an incentive for each robot to move as fast as possible, the competing objectives of preventing load rotation and transporting the load quickly will cause the speeds of the faster team members to oscillate around the speed of the slowest member when it is moving at its maximum speed.

**Figure 5.**
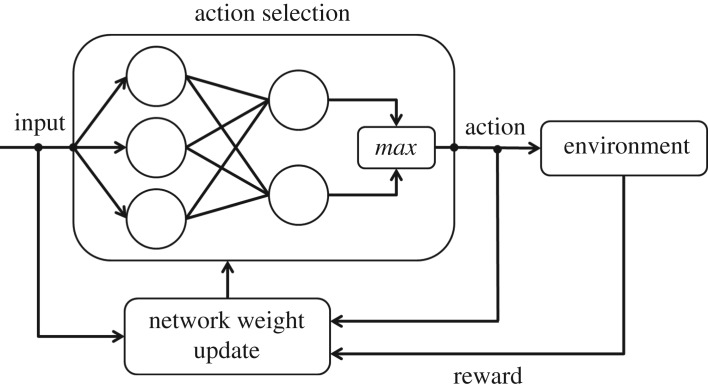
Block diagram of the neural network run by each robot.

A real-time reinforcement learning algorithm was developed to implement this behaviour on robots. The algorithm drives a team of robots with heterogeneous maximum speeds to transport a load at the speed of its slowest member. Each robot runs the two-layer neural network shown in figure 5. There is no distinction between a learning phase and an exploitation phase; instead, an ε-greedy algorithm is chosen to adapt to the unpredictable and changing pulling forces during transport. This is done to allow learning errors that can be made during the start of the transport to be corrected.

For this application, the goal is for the robots to learn the association between the direction of the load’s rotation and the necessary adjustments to the individual speeds of the transporters to reject this rotation and keep the load at its initial orientation. This learning is done at discrete times, where, for simplicity, the times are defined in unit increments. The input vector to the neural network of robot *k* at time *t*, $p_{k}(t)={\left[p_{k,1}\;p_{k,2}\;p_{k,3}\;p_{k,4}\right]}^{T}={\left[\text{sign}(\theta )\;\text{sign}(\dot{\theta })\;\text{sign}(\ddot{\theta })\;\text{sign}({\dot{v}}_{k})\right]}^{T}$, contains the directions of the load’s orientation *θ*, angular velocity $\dot{\theta }$, and angular acceleration $\ddot{\theta }$, as well as the direction of the change in the robot’s velocity ${\dot{v}}_{k}$, all at time *t*. The output vector of the neural network of robot *k* is defined as **a**_*k*_ = [*a*_*k*,1_ *a*_*k*,2_] ^T^ = [*v*_*k*+_ *v*_*k*−_] ^T^, whose entries indicate a binary decision for the robot to speed up or slow down. Each robot *k* computes the value *a*_*k*,*j*_(*t*) of its output neuron *j* ∈ {1, 2} at time *t* as

4.1 $$a_{k,j}(t)=\sum_{i=1}^{4}w_{k,ij}(t)p_{k,i}(t),$$

where *w*_*k*,*ij*_(*t*) is the weight value of the connection between the robot’s *i*th input neuron and *j*th output neuron.

An *ε*-greedy algorithm is applied to determine the action taken by the robot. With probability *ε*, the robot randomly chooses to speed up or slow down without using the neural network to make a decision. Otherwise, the output neuron with the larger value is chosen as the winner. If *v*_*k*+_ ≥ *v*_*k*−_, then robot *k* speeds up; otherwise, it slows down, according to the following controller:

4.2 $${\dot{v}}_{k}=\left\{\begin{array}{cc} K_{a}\frac{\left|\theta \;\right|}{\theta_{\max}}+K_{v}\left(1-\frac{v_{k}}{v_{k,\max}}\right), & \;v_{k+}\geq v_{k-}\text{ or }F_{k}=0\\ -K_{a}\frac{\left|\theta \;\right|}{\theta_{\max}}-K_{v}\left(1-\frac{v_{k}}{v_{k,\max}}\right), & \text{ otherwise }, \end{array}\right.$$

where *K*_*a*_ and *K*_*v*_ are constant gains, *θ*_max_ = *π*, *v*_*k*,max_ is the robot’s maximum possible towing speed, and *F*_*k*_ is the magnitude of the pulling force applied by the robot. When the robot measures *F*_*k*_ = 0 during some time period, it speeds up but does not update its weights *w*_*k*,*ij*_ (learn) during that period.

The velocity controller in equation (4.2) drives inherently faster robots with higher maximum speeds to be more agile than slower robots, since the quantity (1 − *v*_*k*_/*v*_*k*,max_) is larger for faster robots for a given robot speed *v*_*k*_, producing a higher acceleration. This component of the controller prevents the robots from all making the decision to change their transport speeds by the same amount, which would keep their relative speeds constant and, therefore, maintain the direction and speed of the load’s rotation. The robots would not learn anything substantial in this scenario, since any action they take would not affect the load’s rotation and every decision would be penalized according to the reward function described in the next paragraph, even if it were the correct one. The controller also makes larger adjustments to the robots’ accelerations when the payload is far from its initial orientation. The gains *K*_*a*_ and *K*_*v*_ weight the competing objectives of maintaining the load in its original orientation and moving the load as fast as possible.

After taking an action of speeding up or slowing down, each robot computes a reward that is based on the resulting change in the load’s orientation. The reward function at time *t* for robot *k* is defined as

4.3 $$E_{k}(t)=\left\{\begin{array}{cc} \frac{\theta_{\max}-|\theta \;|}{\theta_{\max}}+\frac{v_{k}}{v_{k,\max}}, & \text{sign}(\theta \dot{\theta })\leq 0,\\ -\left|\frac{\theta }{\theta_{\max}}\right|,\; & \text{ otherwise.} \end{array}\right.$$

The reward function can be interpreted as follows. When a robot’s action causes the load to rotate toward its initial orientation $\left(\text{sign}(\theta \dot{\theta })<0\right)$, return to its initial orientation (*θ* = 0), or stop rotating $\left(\dot{\theta }=0\right)$, the robot is rewarded based on the ratio between its current and maximum speeds and the discrepancy that it measures between the load’s current and initial orientations. The robot is penalized based on this discrepancy if its action causes the load to rotate away from its initial orientation $\left(\text{sign}(\theta \dot{\theta })>0\right)$. The difference in reward values *E*_*k*_(*t* − 1) and *E*_*k*_(*t*) at times *t* − 1 and *t*, respectively, determines the adjustments to the neural network weights according to the Instar rule [21]. Reward constants *r*_*k*,*j*_(*t*) are defined at time *t* such that only the output neuron associated with the chosen action is rewarded or penalized:

4.4 $$r_{k,j}(t)=\left\{\begin{array}{cc} 1, & E_{k}(t)-E_{k}(t-1)\geq \tau \text{ and }a_{k,j}>a_{k,l},\;\;l\neq j\\ -1, & E_{k}(t)-E_{k}(t-1)\leq -\tau \text{ and }a_{k,j}>a_{k,l},\;l\neq j\\ 0, & \text{ otherwise},\;\;\;\;\; \end{array}\right.$$

where *τ* is a threshold value chosen to differentiate between sensor noise and a significant reward change. Then the weights are updated as follows:

4.5 $$w_{k,ij}(t)=(1-\gamma r_{k,j}(t))w_{k,ij}(t-1)+\alpha r_{k,j}(t)p_{k,i}(t),$$

where *γ* ∈ [0, 1] is a rate of forgetting and *α* ∈ [0, 1] is a rate of learning. These rates are chosen to bound the maximum weight values of the update matrix to

4.6 $$w_{k,ij}^{\max}=\frac{\alpha }{\gamma }.$$

**Figure 6.**
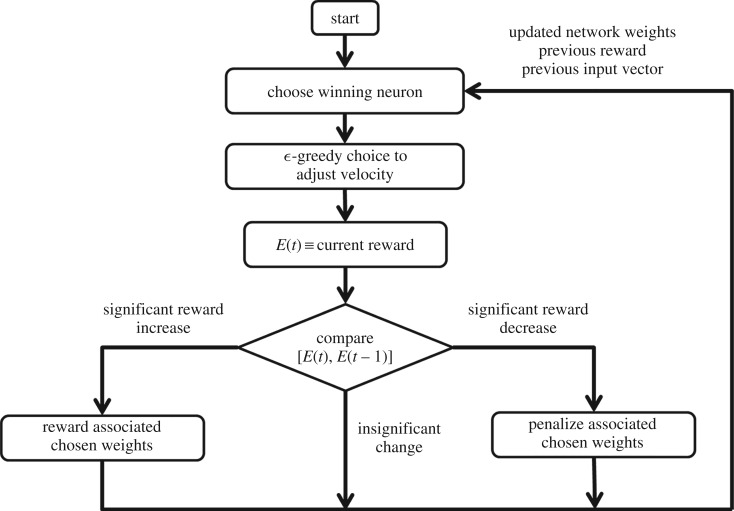
Flowchart of an individual robot’s decision process during transport.

After updating the weights *w*_*k*,*ij*_, the process is repeated. A flowchart of the robot behaviour is shown in figure 6.

## 5. Multi-robot experimental results

### 5.1. Continuous, adaptive pulling

The learning algorithm described in §4 was implemented on teams of two to four Pheeno robots to investigate whether learning through stigmergic feedback could cause a transport team to tow the payload at the speed of the slowest member. In these experiments, the mass of the load was maintained at 500 g, which a single robot is capable of towing. This was done to ensure that the decrease in payload speed could be attributed entirely to the learning algorithm, not to the experimental set-up. To imitate the ants’ directionality of transport toward the nest, all the robots were assigned to drive in the same direction. The robots changed their speed according to the learning algorithm at a rate of 0.5 Hz. Each robot was programmed with a probability *ε* = 0.2 of choosing a random action, a forgetting rate *γ* = 0.02, a learning rate *α* = 0.1, a significance threshold value τ = 0.5, and controller gains *K*_*a*_ = 1 and *K*_*v*_ = 0.8. Ten trials were run for each team size. Teams of two, three and four robots consisted of members with maximum speeds of [4, 12] cm s^−1^, [4, 8, 12] cm s^−1^ and [4, 6, 9, 12] cm s^−1^, respectively. The locations of the robots on the load were chosen randomly for each trial.

**Figure 7.**
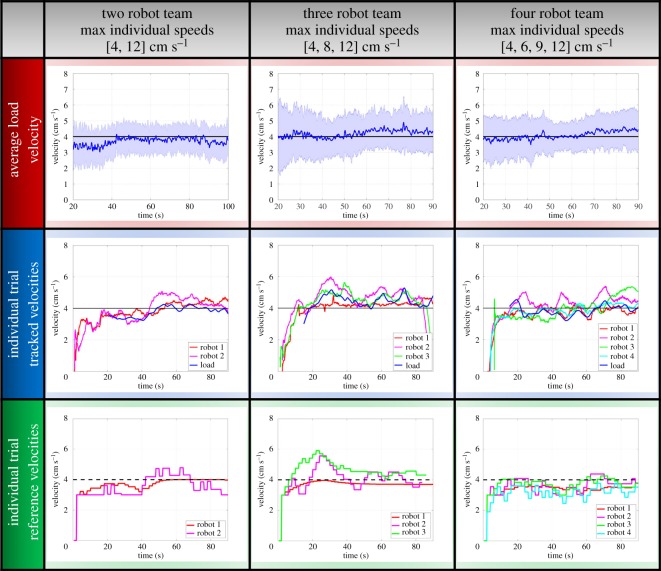
Results of towing experiments with the reinforcement learning algorithm. The plots display the time evolution of the load and robot velocities for each team size. In all plots, the black line is the maximum reference speed of the slowest team member. The first row shows the average load velocity across ten trials, with the solid blue line and shaded area representing the mean and 95% confidence interval, respectively. The second row shows the measured velocities of the robots and the load during a single experimental trial, and the third row shows the corresponding reference velocity that was calculated by each robot using the learning algorithm.

The load and robot velocities during the experiments are plotted in figure 7. These results show that the robots adjust their speed to the speed of the slowest transporter in the team, which moves at a maximum speed of 4 cm s^−1^. For all three team sizes, the 95% confidence interval for mean speed includes the value 4 cm s^−1^ throughout the duration of towing. There are slight discrepancies between the robots’ reference velocities and their actual velocities, which were measured from the robot locations tracked by the overhead camera. These differences were caused by camera error, wheel slip and tag placement error.

### 5.2. Intermittent, constant pulling

To simulate out-of-phase stepping during transport as described in §3.2, towing experiments with two to four Pheeno robots were run in which the robots’ motors were periodically turned on and off to mimic the contact and swing phases of an ant’s gait. The robots’ motors were turned on for 1.5 s during a total step time of 3 s. The robots’ start times for each step were randomly drawn from a uniform distribution. Each robot ‘stepped’ with the same maximum velocity of 8 cm s^−1^. The load mass was scaled with the number of robots in the team, with a constant mass of 500 g per robot. Ten trials were run for each team size.

**Figure 8.**
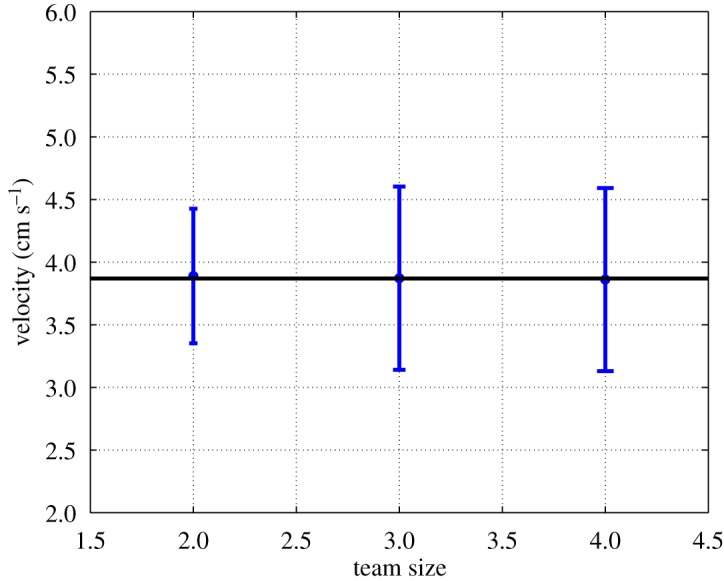
Results of towing experiments with out-of-phase robot stepping. Blue dots with error bars are the mean ± standard deviation of the average speed of the payload across 10 trials. The black line is the prediction of model equation (3.11) of the average steady-state transport speed.

Figure 8 shows that the average steady-state payload speed during these experiments matches the value predicted by the model equation (3.11). There was no significant difference in speed among teams of different size (ANOVA: *F*_2,27_ = 0.017, *p* = 0.98). Thus, it can be concluded that out-of-phase stepping is not the primary cause of the decrease in steady-state transport speed with respect to team size that is observed in *N. cockerelli*.

In addition, out-of-phase stepping was tested on teams of Pheeno robots with different maximum speeds. Under these conditions, the robot teams exhibited uncoordinated transport in which the load was pulled into the slowest robot, which was then dragged along by the efforts of the other robots. This type of behaviour was never observed during the ant towing experiments. Wheel slip ultimately stalled the robots or made them uncontrollable and prevented further transport of the load.

## 6. Discussion

The statistical analysis in §3.1 of data on collective towing by *Novomessor cockerelli* ants and the results of the multi-robot towing experiments described in §5.1 support the conclusion that ants adjust their speeds during collective transport to accommodate the slowest member of the transport team. Hence, robotic implementation of ant-like transport strategies could enable adaptive, decentralized transport by teams of robots with heterogeneous capabilities, manufacturing variations, differences in charge state and other dissimilar properties.

Previous studies have addressed other scenarios where group efficiency is a sublinear function of group size. Krieger *et al.* found that in a group of robots mimicking an ant foraging behaviour, the contribution of each individual to the foraging process decreased as the group size increased due to more time spent avoiding collisions [22]. Lerman & Galstyan also discovered this sublinear relationship in foraging behaviours and predicted an optimal group size for foraging before interference among individuals begins to lower the net group performance [23]. In contrast to these group foraging examples, the sublinear relationship between transport speed and team size that has been investigated here appears to be caused by heterogeneity of teammate capabilities rather than interference arising from uncoordinated individual behaviours (e.g. out-of-phase stepping, unaligned pulling angles or avoidance of fellow transporters).

The differences in individual *N. cockerelli* speeds could be due to variation among ants in fatigue, health or age. However, they may also reflect behavioural differences with implications for colony function [24]. Behavioural disparities may arise from genetic variation among ants or from differences in nutrition, developmental environment or experience. Individual differences can also be amplified via positive feedback generated by social interactions among colony members [25]. Potential benefits of interindividual variation include improved resistance to pathogens and parasites, more efficient allocation of distinct tasks among workers, and more robust response to perturbations [26,27]. For example, honeybee colonies do better at keeping nest temperature within optimal bounds when their workers vary in the temperature threshold that triggers thermoregulatory behaviour [28]. Whether differences in speed among individual *N. cockerelli* ants have similar effects on colony function remains to be investigated.

Cooperative transport allows colonies to harvest large food items that would otherwise require dissection into pieces small enough for single ants to carry [11]. In some ant species, particularly *Eciton burchellii* and other swarm raiders, transport has the further advantage of being *superefficient*. That is, groups can carry more weight per ant than solitary transporters, without any loss in speed [1,29]. This property helps swarm raiders achieve the high rates of food capture needed to support their large, fast-growing colonies. *N. cockerelli*, in contrast, have much smaller colonies and workers forage largely on their own. They also compete with mass-recruiting ant species, such as *Forelius foetidus* and *Solenopsis xyloni*, that can readily displace them from rich food items [10]. This competition may not afford *N. cockerelli* the opportunity to wait for the fastest foragers to retrieve a food item before it is claimed by other ants.

A heterogeneous transport team that adapts to its slowest member will move the load more slowly as the team size increases; however, this strategy also has various benefits. If this adaptation did not occur, the fastest individuals would need to overwhelm the efforts of the slowest members and handle a heavier portion of the load, possibly dragging the slower teammates during the transport. By accommodating the slowest member, large transport teams can use the strength of all teammates, not just the fastest ones and thus can transport heavier loads. Another advantage may arise when the load must be transported over different types of terrain. A large transport team could apply a high net force to a load in order to pull it up a hill or over an obstacle. The presence of slow members in the team could potentially stabilize this manoeuvre, since from a control-theoretic perspective, less aggressive systems are easier to control. Therefore, an ant-like strategy could potentially increase the robustness of the transport to changing, unanticipated environmental conditions.

## 7. Conclusion

In this work, we have investigated the observation that the transport speed of a load towed by several *Novomessor cockerelli* ants decreases as a function of the team size, even with the same load mass per ant. A control approach in which a homogeneous team of robots pull on the load with intermittent, constant forces was tested as a possible ant towing behaviour. A dynamical model was used to predict the steady-state speed of a load that is transported in this manner by a team of known size. The predicted load speed was independent of team size, as long as the mass per transporter remained constant, and the prediction was verified through experiments with teams of two to four small mobile robots. Using order statistics, it was found that the load speed decrease can be attributed to the heterogeneous abilities of individual ants. Since ants within a given colony may move at a range of possible speeds, as a result of differences in characteristics such as age, energy and ability to orient during transport, it was hypothesized that an ant transport team must move at the speed of the slowest member for successful load transport. Owing to their biological limitations, the ants would need to identify the slowest member without explicit communication or global information. To implement a multi-robot towing strategy with these constraints, a real-time reinforcement learning algorithm was developed that relies on implicit communication through the load and local measurements by each robot. This algorithm was tested on teams of two to four robots with significantly different individual maximum speeds. The experimental results supported the hypothesis that the ants are towing the load at the speed of the slowest team member.

The experiments on collective transport described in this paper have focused on one-dimensional payload transport by both ants and robots in flat environments with no obstacles. In the future, experiments and analyses should be conducted for transport along two-dimensional trajectories through more complex environments. To more closely emulate the ant behaviours, multi-robot experiments can be performed using legged robots that are closer in design to the ants’ anatomy. In addition, a rigorous analysis of the reinforcement learning algorithm presented in this work is needed to characterize the existence and stability of equilibrium payload speeds. This analysis would provide theoretical guarantees on the transport dynamics, and therefore enable more confidence in the algorithm’s effectiveness in a wide range of scenarios.

## Supplementary Material

Explanation of the assumption the ant data is normally distributed for proper order statistic analysis.

## Data accessibility

The data from the robot experiments, comprising the robot input data and overhead camera tracking data, can be accessed through the Autonomous Collective Systems Laboratory Github Repository at: https://github.com/ACSLaboratory/robot_towing_data_set/. The data from the ant experiments associated with this paper can be found with the publication Buffin *et al.* [13], which further explains these data.

## References

[1] Czaczkes TJ, Ratnieks FLW 2013 Cooperative transport in ants (Hymenoptera: Formicidae) and elsewhere. Myrmecol. News 18, 1–11.

[2] McCreery HF, Breed MD 2014 Cooperative transport in ants: a review of proximate mechanisms. Insectes Soc. 61, 99–110. (10.1007/s00040-013-0333-3)

[3] Grassé P-P 1959 La reconstruction du nid et les coordinations interindividuelles chez *Bellicositermes natalensis* et *Cubitermes* sp. la théorie de la stigmergie: Essai d’interprétation du comportement des termites constructeurs. Insectes Soc. 6, 41–80. (10.1007/BF02223791)

[4] Ronald Kube C, Bonabeau E 2000 Cooperative transport by ants and robots. Robot. Auton. Syst. 30, 85–101. (10.1016/S0921-8890(99)00066-4)

[5] Ferrante E, Brambilla M, Birattari M, Dorigo M 2013 Socially-mediated negotiation for obstacle avoidance in collective transport. In *Distributed autonomous robotic systems*, pp. 571–583. Berlin, Germany: Springer.

[6] Lindsey QJ, Shomin M, Kumar V 2010 Cooperative quasi-static planar manipulation with multiple robots. In *ASME Int. Design Engineering Technical Conf. and Computers and Information in Engineering Conf., Istanbul, Turkey, 12–14 July*, pp. 1351–1360. American Society of Mechanical Engineers.

[7] Wang Z, Schwager M 2016 Multi-robot manipulation without communication. In *Distributed autonomous robotic systems* (eds N-Y Chong, Y-J Cho), pp. 135–149. Tokyo, Japan: Springer.

[8] Chen J, Gauci M, Groß R 2013 A strategy for transporting tall objects with a swarm of miniature mobile robots. In *Proc. of the IEEE Int. Conf. on Robotics and Automation (ICRA), Karlsruhe, Germany, 6–10 May*, pp. 863–869. IEEE.

[9] Becker A, Habibi G, Werfel J, Rubenstein M, McLurkin J 2013 Massive uniform manipulation: controlling large populations of simple robots with a common input signal. In *Proc. of the IEEE/RSJ Int. Conf. on Intelligent Robots and Systems (IROS), Tokyo, Japan, 3–7 November*, pp. 520–527 IEEE.

[10] Hölldobler B, Stanton RC, Markl H 1978 Recruitment and food-retrieving behavior in *Novomessor* (Formicidae, Hymenoptera: I. Chemical signals). Behav. Ecol. Sociobiol. 4, 163–181. (10.1007/BF00354978)

[11] Buffin A, Pratt SC 2016 Cooperative transport by the ant *Novomessor cockerelli*. Insectes Soc. 63, 429–438. (10.1007/s00040-016-0486-y) PMC6177163 30300423

[12] Kumar GP, Buffin A, Pavlic TP, Pratt SC, Berman S 2013 A stochastic hybrid system model of collective transport in the desert ant *Aphaenogaster cockerelli*. In *Proc. of the 16th Int. Conf. on Hybrid Systems: Computation and Control (HSCC), Philadelphia, PA, 8–11 April*, pp. 119–124. New York, NY: ACM.

[13] Buffin A, Sasaki T, Pratt SC In press. Scaling of speed with group size in cooperative transport by the ant *Novomessor cockerelli* PLoS ONE. 10.1371/journal.pone.0205400 PMC6177163 30300423

[14] Schwarz S, Mangan M, Zeil J, Webb B, Wystrach A 2017 How ants use vision when homing backward. Curr. Biol. 27, 401–407. (10.1016/j.cub.2016.12.019) 28111152

[15] Wilson S, Gameros R, Sheely M, Lin M, Dover K, Gevorkyan R, Haberland M, Bertozzi A, Berman S 2016 Pheeno, a versatile swarm robotic research and education platform. IEEE Robot. Autom. Lett. 1, 884–891. (10.1109/LRA.2016.2524987)

[16] David HA, Nagaraja HN 2005 Expected values and moments. In *Order statistics*, pp. 33–58. New York, NY: John Wiley & Sons, Inc.

[17] Rubenstein M, Cabrera A, Werfel J, Habibi G, McLurkin J, Nagpal R 2013 Collective transport of complex objects by simple robots: theory and experiments. In *Proc. of the Int. Conf. on Autonomous Agents and Multi-Agent Systems (AAMAS), St. Paul, MN, 6–10 May* Richland, SC: International Foundation for Autonomous Agents and Multiagent Systems.

[18] Christensen DL, Suresh SA, Hahm K, Cutkosky MR 2016 Let’s all pull together: principles for sharing large loads in microrobot teams. IEEE Robot. Automat. Lett. 1, 1089–1096. (10.1109/LRA.2016.2530314)

[19] Peshkin MA, Sanderson AC 1989 Minimization of energy in quasi-static manipulation. IEEE Trans. Robot. Autom. 5, 53–60. (10.1109/70.88017)

[20] Trinkle JC 1989 A quasi-static analysis of dextrous manipulation with sliding and rolling contacts. In *Proc. of the IEEE Int. Conf. on Robotics and Automation (ICRA) Scottsdale, AZ, 14–19 May*, pp. 788–793. IEEE.

[21] Hagan MT, Demuth HB, Beale MH, De Jesús O 2014 Neural network design, 2nd edn Boston, MA: PWS Publishing Co.

[22] Krieger MJB, Billeter J-B, Keller L 2000 Ant-like task allocation and recruitment in cooperative robots. Nature 406, 992–995. (10.1038/35023164) 10984052

[23] Lerman K, Galstyan A 2002 Mathematical model of foraging in a group of robots: effect of interference. Autonom. Robots 13, 127–141. (10.1023/A:1019633424543)

[24] Jandt JM, Bengston S, Pinter-Wollman N, Pruitt JN, Raine NE, Dornhaus A, Sih A 2014 Behavioural syndromes and social insects: personality at multiple levels. Biol. Rev. 89, 48–67. (10.1111/brv.12042) 23672739

[25] Jeanson R, Weidenmüller A 2014 Interindividual variability in social insects—proximate causes and ultimate consequences. Biol. Rev. 89, 671–687. (10.1111/brv.12074) 24341677

[26] Mattila HR, Seeley TD 2007 Genetic diversity in honey bee colonies enhances productivity and fitness. Science 317, 362–364. (10.1126/science.1143046) 17641199

[27] Beshers SN, Fewell JH 2001 Models of division of labor in social insects. Annu. Rev. Entomol. 46, 413–440. (10.1146/annurev.ento.46.1.413) 11112175

[28] Jones JC, Myerscough MR, Graham S, Oldroyd BP 2004 Honey bee nest thermoregulation: diversity promotes stability. Science 305, 402–404. (10.1126/science.1096340) 15218093

[29] Franks NR 1986 Teams in social insects: group retrieval of prey by army ants (*Eciton burchelli*, Hymenoptera: Formicidae). Behav. Ecol. Sociobiol. 18, 425–429. (10.1007/bf00300517)

