# Supplementary material for: Multi-robot replication of ant collective towing behaviours
Source: R Soc Open Sci. 2018 Oct 17;5(10):180409. doi: 10.1098/rsos.180409 (PMC6227946; doi:10.1098/rsos.180409)
Supplement: Explanation of the assumption the ant data is normally distributed for proper order statistic analysis. [file rsos180409supp1.pdf]

The order statistics analysis presented in this paper relies on the assumption that the individual transport speeds observed in *Novomessor cockerelli* ants are normally distributed. Here we justify this assumption by applying statistical tests to the data. Figure 1(a) shows a histogram of data from [1] on the transport speeds of individual ants. Both the Anderson-Darling and Kolmogorov-Smirnov tests for normality find no significant departure from normality ($p = 0.05$). The quantile-quantile (q-q) plot in Figure 1(b) also shows an approximately linear relationship. However, this data has a skewness value of 0.4985, which arises due to the inherent lower bound of the data (i.e., the magnitude of the transport speed cannot be negative). This positive skewness is evident in both Figure 1(a) and Figure 1(b).

Although the assumption of normally distributed transport speeds is valid, it is important to check whether our order statistics analysis still predicts the observed relationship between load transport speed and team size from an unskewed version of the data. To eliminate the skew in the data, a Box-Cox transformation [2, 3] is applied to the measured individual transport speeds. This reduces the skewness value of the data to -0.0499. The transformed data with a Gaussian fit is shown in Figure 2(a), with the associated q-q plot shown in Figure 2(b). The figures confirm that the transformed data is no longer subject to the inherent lower bound of the original data. Following the procedure described in Section 3.1, we computed the expected value and standard deviation of the speed of the slowest transporter in a team with two, three, and four members. The probability density function $\phi(v)$ in Equation (3) and Equation (4) was defined as the Gaussian fit to the transformed ant data, shown in Figure 2(a). The computed values of $E(1, n) \pm Var(1, n)^{1/2}$ for $n = 2, 3, 4$ are plotted alongside the statistics of the Box-Cox transformed ant data in Figure 3. The figure shows that the mean and standard deviation of the transformed ant data for each team size are close to those of the corresponding first order statistics. Thus, our order statistics analysis of the transformed data still predicts the observed reduction in steady-state transport speed with increasing team size. Since the original ant data is easier to interpret than the transformed data, the analysis presented in the paper is conducted with the original data.

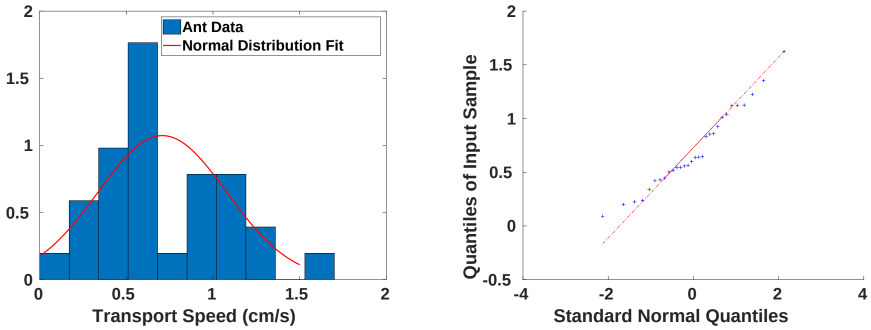

(a) (b)

Figure 1. (a) Histogram (blue) of data on the individual ant transport speeds from *N. cockerelli* experiments [1], with a Gaussian fit (red). (b) Quantile-quantile plot of the same data.

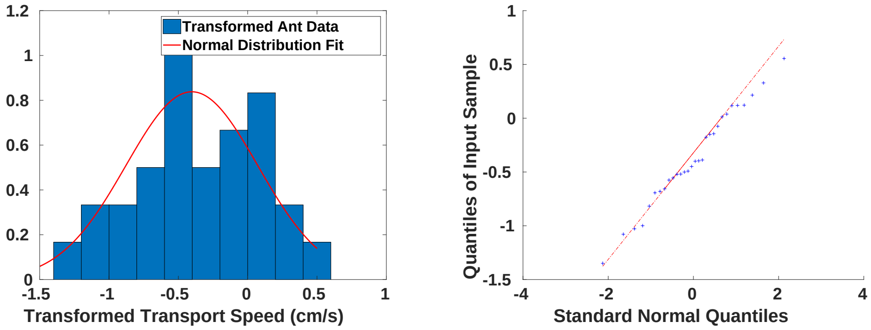

(a) (b)

Figure 2. (a) Histogram (blue) of Box-Cox transformed data on the individual ant transport speeds from *N. cockerelli* experiments [1], with a Gaussian fit (red). (b) Quantile-quantile plot of the same data.

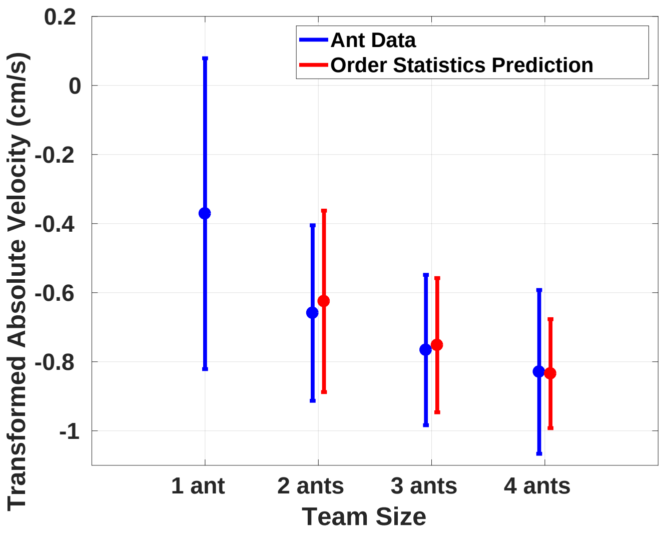

FIGURE 3. *Blue plots:* Box-Cox transformed steady-state speed of an artificial load (0.3 g per ant) towed by different numbers of ants [1]. The circles with error bars represent the mean ± standard deviation across 30 experimental trials (10 trials per ant colony). *Red plots:* Mean ± standard deviation of the first order statistic of $n = 2, 3, 4$ samples from a normal distribution that is fit to individual ant transport speed data under the Box-Cox transformation.

References

[1] Aurélie Buffin, Takao Sasaki, and Stephen C. Pratt. Scaling of speed with group size in cooperative transport by the ant *Novomessor cockerelli*. Submitted to PLOS ONE, 2018.

[2] George E. P. Box and David R. Cox. An analysis of transformations. *Journal of the Royal Statistical Society. Series B (Methodological)*, 26(2):211–252, 1964.

[3] R. M. Sakia. The Box-Cox transformation technique: a review. *Journal of the Royal Statistical Society. Series D (The Statistician)*, 41(2):169–178, 1992.
